# The Selective Serotonin Reuptake Inhibitor Paroxetine, but not Fluvoxamine, Decreases Methamphetamine Conditioned Place Preference in Mice

**DOI:** 10.2174/157015911795017236

**Published:** 2011-03

**Authors:** Y Takamatsu, H Yamamoto, Y Hagino, A Markou, K Ikeda

**Affiliations:** 1 Division of Psychobiology, Tokyo Institute of Psychiatry, 2-1-8 Kamikitazawa, Setagaya-ku, Tokyo 156-8585, Japan; 2 Department of Psychiatry, School of Medicine, University of California San Diego, 9500 Gilman Drive, La Jolla, San Diego, CA 92093-0603, USA

**Keywords:** Conditioned place preference, Fluvoxamine, Methamphetamine, Mice, Paroxetine, Serotonin transporter.

## Abstract

Monoamine transporters are the main targets of methamphetamine (METH). Recently, we showed that fluoxetine, a selective serotonin reuptake inhibitor (SSRI), decreased METH conditioned place preference (CPP), suggesting that serotonin transporter (SERT) inhibition reduces the rewarding effects of METH. To further test this hypothesis, in the present study we investigated the effects of additional SSRIs, paroxetine and fluvoxamine, on METH CPP in C57BL/6J mice. In the CPP test, pretreatment with 20 mg/kg paroxetine abolished the CPP for METH, whereas pretreatment with 100 mg/kg fluvoxamine prior to administration of METH failed to inhibit METH CPP. These results suggest that paroxetine, a medication widely used to treat depression, may be a useful tool for treating METH dependence. Further, these data suggest that molecules other than the SERT [such as G protein-activated inwardly rectifying K+ (GIRK) channels] whose activities are modulated by paroxetine and fluoxetine, but not by fluvoxamine, are involved in reducing METH CPP by paroxetine and fluoxetine.

## INTRODUCTION

Methamphetamine (METH) is abused in worldwide [1]. In Japan, the number of people arrested for METH possession or use is approximately 100 times higher than those arrested for cocaine, opioids, or cannabis. Further, METH frequently induces psychotic states with symptoms similar to those seen in paranoid schizophrenia [2]. Such psychotic states are treated primarily in hospitals resulting in high medical costs. Thus, there is great need for the discovery of new medications for METH abuse [3] because the current treatments are mostly oriented toward the treatment of psychosis with no treatments available to prevent relapse to METH abuse.

The dopamine transporter (DAT) is the main target for METH and cocaine. However, mice lacking the DAT show conditioned place preference (CPP) to cocaine [4] and self-administer cocaine [5]. Interestingly, heterozygous and homozygous serotonin transporter (SERT) knockout mice that also have a homozygous knockout of the DAT do not exhibit cocaine CPP [6]. Cocaine administration leads to increases in extracellular dopamine concentration in the striatum of DAT knockout mice but not of DAT/SERT double knockout mice [7]. Taken together, these reports suggest that SERT inhibition may decrease METH and cocaine CPP.

Recently, we showed that fluoxetine, a selective serotonin reuptake inhibitor (SSRI), abolished METH CPP when METH was administered during both the development and expression phases of the CPP procedure, supporting the hypothesis that SERT inhibition decreased the rewarding effects of METH [8]. To further test this hypothesis, in the present study we investigated the effects of the SSRIs paroxetine (Paxil^®^) and fluvoxamine (Lubox^®^ or Depromel^®^) on METH CPP.

## MATERIALS AND METHODS

### Mice

Male C57BL/6J mice (8-10 weeks old) were purchased from CLEA Japan, Inc. (Tokyo, Japan) and were housed for 1-2 weeks before the experiments began in an animal facility maintained at 22 ± 2˚C and 55 ± 5% relative humidity under a 12/12 h light/dark cycle with lights on at 8:00 am. Food and water were available *ad libitum.* All behavioral testing was conducted during the light phase. The experimental procedures and housing conditions were approved by the Institutional Animal Care and Use Committee of the Tokyo Institute of Psychiatry, and all animals were cared for and treated humanely in accordance with our institutional animal experimentation guidelines.

### Conditioned Place Preference (CPP) Test

The CPP test was performed according to the method of Hoffman and Beninger [9] with some modifications. We used a two-compartment Plexiglas chamber (Neuroscience Inc., Osaka, Japan). One compartment (17.5 × 15 × 17.5 cm: width × length × height) was black with a smooth floor, and the other compartment was of the same dimensions, but with a white textured floor. This two-compartment chamber was located in a sound- and light-attenuated box under conditions of dim illumination (approximately 40 lux) to reduce bias toward either compartment [10]. Mice were assigned randomly to the treatment groups (see below).

On Day 1, the mice (*n* = 14-26 per group) were allowed to freely explore the two compartments for 15 min. On Day 2, the mice again were allowed to explore the two compartments freely for 15 min, and the time spent in each compartment and the number of transitions between compartments were measured. Conditioning sessions then were conducted once daily for 4 consecutive days (Days 5-8). For the Day 5 conditioning session, mice were i.p. injected with saline or SSRI (20 mg/kg paroxetine or 100 mg/kg fluvoxamine) 60 min before injection with METH (2 mg/kg, i.p.). Immediately after METH administration, mice were confined to the black or white compartment for 50 min. On Day 6, the mice were pretreated with the same solution (saline or SSRI, i.p.) 60 min before a saline injection. Immediately after the saline injection, mice were confined to the opposite compartment for 50 min. On Days 7 and 8, the same conditioning as on Days 5 and 6 was repeated. On Day 9, the mice were pretreated with saline or SSRI (20 mg/kg paroxetine or 100 mg/kg fluvoxamine, i.p.), and 60 min later were allowed to freely explore the two compartments for 15 min without METH injection. The time spent in each compartment and the number of transitions between compartments were measured. In summary, there were a total of eight groups in this experiment corresponding to the four pretreatments (paroxetine, fluvoxamine, saline; there were two saline groups that were run concurrently with the paroxetine and fluvoxamine groups) and the two phases of the experiment during which they were pretreated with the drug (conditioning days 5-8 or test day 9). The CPP score was defined as the time spent in the drug-paired compartment during the CPP test phase (Day 9) minus the time spent in the same compartment during the preconditioning exploratory phase (Day 2). The transition score was defined as the number of transitions during the CPP test phase (Day 9) minus the number of transitions during the preconditioning exploratory phase (Day 2).

### Drugs

Methamphetamine hydrochloride was purchased from Dainippon Pharmaceutical (Osaka, Japan). Paroxetine maleate and fluvoxamine maleate were purchased from Sigma (St. Louis, MO, USA) and TOCRIS (Hung Road, Bristol, UK), respectively. All drugs were dissolved in saline. Drugs and vehicle were administered i.p. in a volume of 0.1 ml/10 g body weight. All drug doses are reported as salt.

### Statistical Analyses

The CPP and transition scores of mice pretreated with saline or SSRI during the conditioning and CPP test phases were subjected to a two-way analysis of variance (ANOVA). The ANOVA had two between-subjects factors, each with two levels (saline/SSRI pretreatment in the conditioning phase and saline/SSRI pretreatment in the CPP test phase). Two separate ANOVAs were conducted on the paroxetine and fluvoxamine data. Similar ANOVAs were conducted on the transition scores. The CPP scores from the paroxetine experiment were subjected to a one-way ANOVA followed by *post hoc* comparisons with the Scheffe test. In this ANOVA, there were four levels corresponding to the four treatment conditions (saline in both the conditioning and the CPP test phases, pretreatment with paroxetine only in the conditioning phase, pretreatment with paroxetine only in the CPP test phase, pretreatment with paroxetine in both the conditioning and the CPP test phases). For the CPP data, the durations of time that the mice spent in the METH-paired compartment before and after conditioning were compared using paired *t*-tests for each group. For the transition data, the number of transitions between the METH-paired compartment and the saline-paired compartment before and after conditioning were compared using paired *t*-tests for each group. The level of significance was set at 0.05.

## RESULTS

### Effects of Paroxetine on METH CPP

The two-way ANOVA revealed that mice treated with paroxetine during the test phase exhibited decreased CPP scores compared to mice treated with saline during the test phase (*F*_1,72_ = 7.888, *P* < 0.01), whereas mice treated with paroxetine during the conditioning phase did not differ significantly from mice treated with saline during the test phase in the CPP score [*F*_1,72_ = 1.704, not significant (n.s.); Fig. (**1A**)]. There was no statistically significant interaction between the factor saline/paroxetine during the conditioning phase and the factor saline/paroxetine during the CPP test phase (*F*_1,72_ = 0.1690, n.s.), indicating that the important factor was treatment with paroxetine during the expression phase of the experiment. In addition, a one-way ANOVA on the CPP scores was conducted on data for all four groups. The ANOVA showed a significant difference in the CPP scores among these four groups (*F*_3,72_ = 3.940, *P * < 0.05). The Scheffe *post hoc* test showed that the CPP score of the paroxetine/paroxetine group was significantly lower than that of the saline/saline group (*P* < 0.05). Paired *t*-tests were conducted to compare the duration of time before and after conditioning for each of the four groups (Fig. (**1B**)). Whereas the saline/saline and paroxetine/saline groups spent significantly more time in the METH-paired compartment after conditioning than before conditioning (saline/saline: *n* = 23, *df* = 22, *t* = -6.050, *P* < 0.001; paroxetine/saline: *n* = 15, *df* = 14, *t* = -2.884, *P* < 0.05), the saline/paroxetine and paroxetine/paroxetine groups did not show METH CPP (saline/paroxetine: *n* = 15, *df* = 14, *t* = -2.033, n.s.; paroxetine/paroxetine: *n* = 23, *df* = 22, *t* = -0.908, n.s.). Paroxetine pretreatment had no significant effects on the transition scores compared to the saline/saline treatment group (data not shown).

### Effects of Fluvoxamine on the METH CPP

The two-way ANOVA revealed that both the factor saline/fluvoxamine pretreatment during the conditioning phase and the factor saline/fluvoxamine pretreatment during the CPP test phase had no effects on CPP scores (conditioning phase: *F*_1,68_ = 0.045, n.s.; CPP test phase: *F*_1,68_ = 3.016, n.s.; Fig. (**2A**)). There was no statistically significant interaction between the two factors (*F*_1,68_ = 0.066, n.s.). Paired *t*-tests were conducted to compare the duration of time before and after conditioning for each of the four groups. All four groups spent significantly more time in the METH-paired compartment after conditioning than before conditioning (saline/saline: *n* = 26, *df* = 25, *t *= -4.541, *P* < 0.001; saline/ fluvoxamine: *n* = 14, *df* = 13, *t* = -2.983, *P* < 0.05; fluvoxamine/ saline: *n* = 18, *df* = 17, *t* = -3.949, *P* < 0.01; fluvoxamine/ fluvoxamine: *n* = 14, *df* = 13, *t* = -2.757, *P* < 0.05).

The two-way ANOVA revealed that both fluvoxamine pretreatment during the conditioning phase and during the CPP test phase significantly decreased transition scores (conditioning phase: *F*_1,68_ = 24.321, *P* < 0.001; CPP test phase: *F*_1,68_ = 10.292, *P* < 0.01; Fig. (**2B**)). There was no statistically significant interaction between the two factors (*F*_1,68_ = 0.007, n.s.). Paired *t*-tests were conducted to compare the number of transitions before and after conditioning for each of the four groups. The S-S group showed no significant differences in the number of transitions before and after conditioning (*n* = 26, *df* = 25, *t* = -1.213, n.s.). However, mice pretreated with fluvoxamine (saline/fluvoxamine, fluvoxamine/saline, fluvoxamine/fluvoxamine) showed significant decreases in the number of transitions after conditioning (saline/fluvoxamine: *n* = 14, *df* = 13, *t* = 3.829, *P* < 0.01; fluvoxamine/saline: *n* = 18, *df* = 17, *t* = 5.520, *P* < 0.001; fluvoxamine/fluvoxamine: *n* = 14, *df* = 13, *t* = 6.025, *P* < 0.001).

## DISCUSSION

In the present study, we showed that paroxetine, a widely used medication for treating depression, inhibited METH CPP in mice, similar to the results we reported previously with fluoxetine [8]. No significant effects of paroxetine on transition scores suggest that the effects of paroxetine on METH CPP are not due to changes in locomotor activity but due to reduction of METH reward and conditioned reward by paroxetine. Based on these findings, it appears worthwhile to investigate the clinical effects of paroxetine on METH abuse. By contrast, the other SSRI tested here, fluvoxamine, did not affect METH CPP. These data demonstrate that there are differences in the effects of SSRIs on METH CPP, suggesting the possibility that molecules other than the SERT are involved in the inhibition of METH CPP by paroxetine and fluoxetine reported here and in our previous study [8].

In addition to SERT inhibition, paroxetine inhibits the function of muscarinic cholinergic receptors [11], nicotinic acetylcholine receptors [12], volume-related anion channels [13], membrane steroid transporters [14], and nitric oxide synthase [15]. Recently, Kobayashi and colleagues [16] reported that paroxetine also inhibits the function of G protein-activated inwardly rectifying K^+^ (GIRK) channels. It is intriguing that paroxetine and fluoxetine, but not fluvoxamine, inhibit GIRK channels [16-18]. Various G protein-coupled receptors (such as M2 muscarinic, α2 adrenergic, D_2_ dopaminergic, 5-HT_1A_, opioid, nociceptin/orphanin FQ, and A_1_ adenosine) activate GIRK channels [19-22] through the direct action of G protein subunits [23]. In addition, GIRK channels are activated by ethanol independently of G protein-coupled signaling pathways [24, 25]. Activation of GIRK channels leads to membrane hyperpolarization [22]. These channels play an important role in the inhibitory regulation of neuronal excitability. Thus, modulators of GIRK channel activity may affect many brain functions. Kobayashi and colleagues [26] also have reported that ifenprodil, a cerebral vasodilator which inhibits morphine CPP [27], also inhibits the function of GIRK channels. Morgan and colleagues [28] demonstrated that GIRK channel knockout mice exhibited dramatically reduced intravenous self-administration of cocaine. In the present study, we found that paroxetine and fluoxetine, but not fluvoxamine, inhibited METH CPP. These findings, together with the previous findings, suggest that the inhibition of GIRK channels by paroxetine or fluoxetine may be involved in the inhibition of METH CPP by these drugs.

Fluvoxamine administration (60 mg/kg) leads to a significant decrease in spontaneous locomotor activity [29]. Consistent with this observation, significant decreases in transition scores were observed in all of the 100 mg/kg fluvoxamine-treated groups compared to the saline/saline-treated group in the present study. The number of transitions of the fluvoxamine/fluvoxamine treated group during the CPP test phase (101.4 ± 85.3, mean ± SEM) was the smallest among the four groups in this experiment, but more than 100 transitions indicated adequate locomotion to reveal potential differences in CPP. The lack of effect of fluvoxamine on CPP for methamphetamine is likely to reflect a lack of effect of fluvoxamine on the rewarding effects of METH rather than being a nonspecific effect of fluvoxamine.

In conclusion, we found that paroxetine, but not fluvoxamine, inhibited METH CPP in mice. Although further preclinical studies are needed to elucidate the mechanisms underlying these inhibitory effects of paroxetine on processes relating to METH dependence, it appears worthwhile to investigate the clinical effects of paroxetine on METH abuse. The present results suggest that molecules other than the SERT (such as GIRK channels) are involved in the inhibition of METH CPP by paroxetine and fluoxetine.

## Figures and Tables

**Fig. (1):**
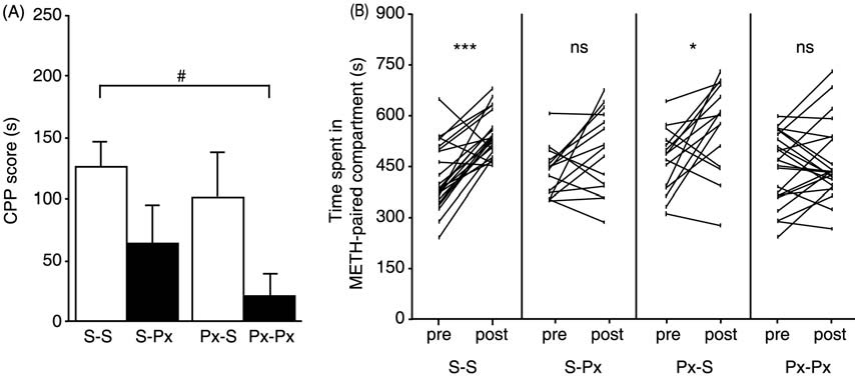
Effects of paroxetine on CPP for METH in mice. (**A**) Reduction of METH CPP by paroxetine (Px) pretreatment. Mice were pretreated with saline (S) in both the conditioning and CPP test phases (S-S), paroxetine only in the CPP test phase (S-Px), paroxetine only in the conditioning phase (Px-S), and paroxetine in both the conditioning and the CPP test phases (Px-Px). The CPP score was defined as the time spent in the drug-paired compartment during the CPP test phase (Day 9) minus the time spent in the same compartment during the preconditioning phase (Day 2). The CPP score of the Px-Px group was significantly lower than that of the S-S group (^#^*P* < 0.05). (**B**) Comparison of time spent in the conditioned compartment before and after conditioning in the four groups. There was a significant CPP in the S-S and Px-S groups, but not in the S-Px and Px-Px groups (when paroxetine was administered in the CPP test phase). ***P < 0.001, *P < 0.05, ns: not significant (*P* > 0.05).

**Fig. (2):**
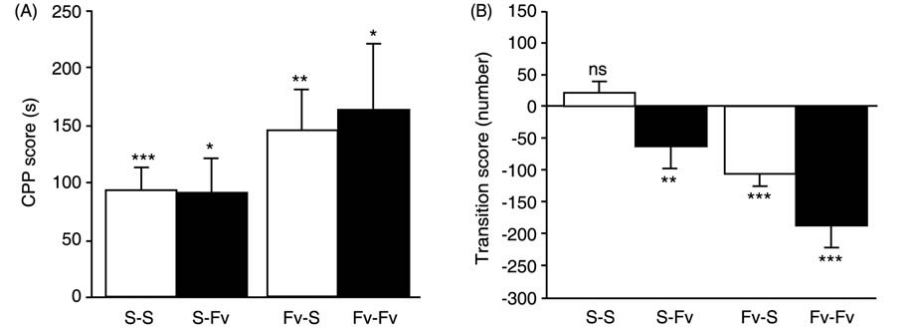
Effects of fluvoxamine on CPP for METH and on transitions between compartments. (**A**) Lack of a significant effect of fluvoxamine (Fv) on METH CPP. Mice were pretreated with saline in both the conditioning and the CPP test phases (S-S), fluvoxamine only in the CPP test phase (S-Fv), fluvoxamine only in the conditioning phase (Fv-S), and fluvoxamine in both the conditioning and the CPP test phases (Fv-Fv). There was a significant CPP in all groups. Fluvoxamine pretreatment in the conditioning phase and/or the CPP test phase failed to inhibit METH CPP (pre- and post-conditioning preference test results were analyzed with paired t-tests, ***P < 0.001, **P < 0.01, *P < 0.05). (**B**) Decreases in transitions between the compartments by fluvoxamine pretreatment. There were significant decreases in transitions in the S-Fv, Fv-S, and Fv-Fv groups, but not in the S-S group [number of transitions in the pre- and post-conditioning phases was analyzed with paired t-tests, ***P < 0.001, **P < 0.01, ns: not significant (*P* > 0.05)]. The transition score was defined as the number of transitions during the CPP test phase (Day 9) minus the number of transitions during the preconditioning phase (Day 2).

## ABBREVIATIONS

ANOVA: = Analysis of variance
CPP: = Conditioned place preference
DAT: = Dopamine transporter
GIRK: = G protein-activated inwardly rectifying K^+^
METH: = Methamphetamine
n.s.: = Not significant
SERT: = Serotonin transporter
SSRI: = Selective serotonin reuptake inhibitor
